# Postnatal temperature triggers predictable thermoregulatory shifts in birds without a trade-off between heat and cold tolerance

**DOI:** 10.1242/jeb.251867

**Published:** 2026-05-13

**Authors:** Elin Persson, Maria Correia, Andreas Nord

**Affiliations:** ^1^ Lund University, Department of Biology, Evolutionary Ecology and Infection Biology, Kontaktvägen 10, SE-223 62 Lund, Sweden; ^2^ University of Jyväskylä, Department of Biological and Environmental Science, Survontie 9, FI-40500 Jyväskylä, Finland; ^3^ Swedish Centre for Impacts of Climate Extremes (climes), Lund University, Lund, Sweden

**Keywords:** Bird, Development, Evaporative cooling, Metabolic rate, Temperature tolerance, Thermoregulation

## Abstract

Thermoregulatory competence is key to maintaining fitness in thermally unstable environments. Previous research found that development in the warmth renders birds better able to handle mild heat stress, whereas cold postnatal development gives no apparent benefit upon mild cold exposure. It is still not known how developmental temperature affects maximal temperature tolerance limits, and even less is known about any physiological trade-off between heat and cold tolerance. We investigated how postnatal development under simulated cold snap or heatwave-like conditions from hatching until reproductive maturity impacted the ontogeny of maximal heat and cold tolerance, and the trade-off between them, in Japanese quail (*Coturnix japonica*). To study whether any such effects are reversible or permanently programmed, we transferred half of each treatment group to intermediate common garden conditions once reproductive maturity was reached and repeated the measurements several weeks later. Development in heatwave-like conditions increased evaporative water loss rate and moved heat tolerance limits upwards, whereas cold snap-like development rendered more thermogenic birds with improved cold tolerance limits. However, we found no evidence for a trade-off between heat and cold tolerance. The common garden birds converged in nearly all thermoregulatory traits at the end of the study, suggesting that the prior emergence of temperature-dependent phenotypes reflected reversible plasticity. We suggest that improved temperature tolerance limits improve performance in matched thermal conditions, reducing the rate at which thermal injury accrues. Yet, in the short term, we found markedly lower capacity to acclimate heat tolerance compared with cold tolerance.

## Teaser

**Summary:** Warm or cold developmental temperatures affect heat and cold tolerance at population levels in Japanese quail; but at individual levels, there was no evidence for a trade-off between tolerance traits.

## INTRODUCTION

Accumulating evidence suggests that both unusually high or low temperatures impact nearly every aspect of performance in both endothermic and ectothermic animals, ranging from offspring growth, maturation and survival (Cunningham et al., 2013; Nord and Nilsson, 2011; Salaberria et al., 2013; De Jong et al., 2023; Noble et al., 2018) to adult behaviour and reproduction (Cunningham et al., 2021; Edwards et al., 2015; Nilsson and Nord, 2018; Nord and Nilsson, 2019; Archer et al., 2025). It follows that high thermoregulatory precision and flexibility is key to maintaining fitness in thermally labile environments. In ostriches (*Struthio camelus*), for example, a more pronounced capacity for heat dissipation leads to higher egg laying rates during hotter conditions compared with less capable heat-dissipating females (Svensson et al., 2023). Similarly, black-capped chickadees (*Poecile atricapillus*) with higher maximal thermogenic capacity had higher probability of overwinter survival compared with less thermogenic birds (Petit et al., 2017). By comparison, ectotherms are predicted to be impacted more strongly by changing long-term or short-term temperature fluctuations on account of their general lack of physiological means for thermoregulation (Kingsolver et al., 2013), particularly in terrestrial habitats (Noble et al., 2025).

The importance of body temperature control in a changing world raises the question of the ontogeny of thermoregulatory phenotypes. It has been hypothesised that thermal conditions during the developmental period may prime subsequent temperature tolerance through developmental plasticity (i.e. a permanent modification of the phenotype triggered by the developmental environment; Piersma and Drent, 2003) acting on key physiological pathways (e.g. hormonal control of thermogenesis and homeostasis; Bize et al., 2010; Piestun et al., 2008). This has often been interpreted as thermal adaptation that increases fitness when the developmental and post-developmental conditions overlap (reviewed by Nichelmann, 2004; Nord and Giroud, 2020), but risks carrying potential costs when these environments are mismatched (Nord and Giroud, 2020). For instance, in the chicken, prenatal heat pulses during the second and third trimester of embryonic development improve postnatal heat tolerance (e.g. Piestun et al., 2011). By comparison, the long-term consequences of postnatal temperature on thermoregulatory traits are not well understood. However, short-term, perinatal heat exposure in chicken has been found to increase survival during heat stress several weeks later (Arjona et al., 1988). On the other hand, research on Japanese quail (*Coturnix japonica*) shows that birds reared under heatwave-like temperatures during the first weeks after hatching were better at handling mild heat stressors (by reducing metabolic heat production and elevating evaporative cooling capacity) as juveniles (Persson et al., 2024), but these effects disappeared when the heat stressor was removed. Analogous exposure to cold snap-like postnatal temperature did not result in any thermoregulatory changes upon exposure to mild cold. These responses probably reflect phenotypic flexibility; that is, reversibly plastic modification of the phenotype mapping environmental variation (Piersma and Drent, 2003). The diversity of responses to postnatal developmental temperatures suggests that more knowledge is needed. For example, it can be speculated that physiological measurements below thermal limits do not capture the full dynamics of how developmental temperature influences heat and cold tolerance.

Almost all avian heat tolerance studies have been performed in hot and dry climates, on the assumption that animals in such biomes have a low capacity to accommodate additional thermal stress. However, even high-latitude birds overheat frequently (e.g. Nilsson and Nord, 2018; O'Connor et al., 2024; Choy et al., 2021) and experimental relief from this risk increases fitness by re-investment of resources otherwise spent on thermoregulation into both current and future reproduction (Andreasson et al., 2020; Nord and Nilsson, 2019). Indeed, the fitness costs of warming may be even more severe at high latitude, where animals are poorly equipped to deal with heat stress (Nord and Folkow, 2018; O'Connor et al., 2021), possibly on account of weak historic selection for high temperature tolerance. In line with this, recent work shows that, in ostriches, there is a negative genetic correlation between heat and cold tolerance at the level of reproductive effort (Schou et al., 2022) that may be further modified by local adaptation of thermoregulation experienced in the natural ranges of different subspecies (Svensson et al., 2023). It has been proposed that improved heat tolerance also decreases cold tolerance, or vice versa, in ectotherms, such that optimization of one trait will hinder the other. For example, studies on fruit flies (*Drosophila melanogaster*) have found evidence for genetic links between heat and cold tolerance (Kristensen et al., 2007; Michalak et al., 2019). These findings across endo- and ectotherms have led some to suggest that constraints on temperature adaptations possibly apply generally across organisms (Schou et al., 2022). Yet, other studies on the fruit fly show that there is a positive correlation between heat and cold tolerance (Bubliy and Loeschcke, 2005), and warm adapted zebrafish (*Danio rerio*) similarly displayed improved cold tolerance (Andreassen et al., 2025). This positive correlation may be less likely to occur in endotherms, because acclimation to high temperature typically involves downregulation of metabolic rate to lessen heat gain (Collin et al., 2001; Wojciechowski et al., 2021; Vages et al., 2024) but upregulation of evaporation to improve heat loss (Talbot et al., 2017), whereas cold acclimation is associated with upregulated heat production rate to support thermogenesis. Yet, to the best of our knowledge, no endotherm study has yet addressed whether the purported trade-off between heat and cold limits has a physiological basis.

**Fig. 1.**
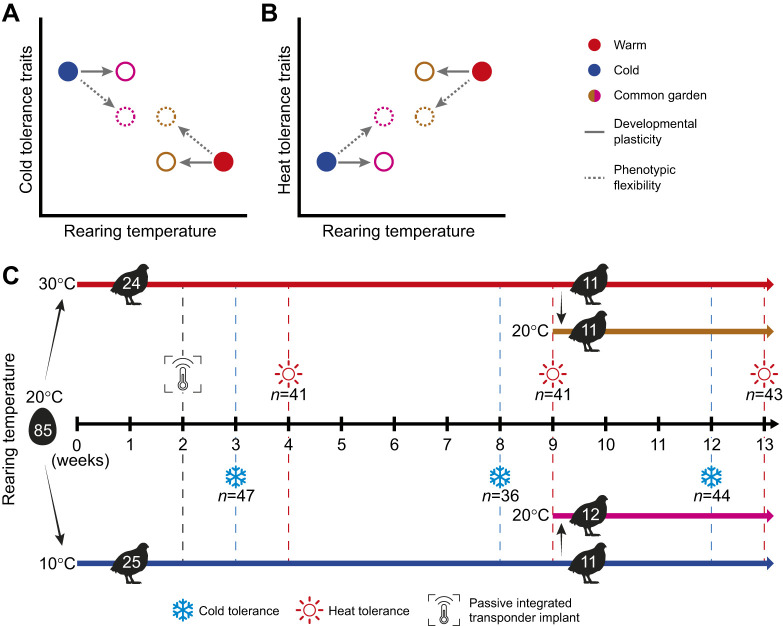
**Predictions and schematic overview of the experimental protocol used to test them.** (A,B) Predicted effects on temperature tolerance traits before and after transfer to common garden-conditions, if postnatal rearing temperature causes developmental plasticity (solid lines and arrows) or phenotypic flexibility (dotted lines and arrows), respectively. (C) Schematic overview of the experimental protocol. Japanese quail were raised in either Warm (30°C) or Cold (10°C) conditions until 9 weeks of age, after which half of each treatment group were transferred to common garden conditions (Cold–mild and Warm–mild; 20°C). Cold tolerance was measured at 3, 8 and 12 weeks and heat tolerance at 4, 9 and 13 weeks. At 2 weeks of age, the birds were implanted with a passive integrated transponder to measure body temperature, as described in the main text.

We investigated whether postnatal developmental temperatures impact the ontogeny of maximal heat- and cold tolerance limits. By measuring empirically the relationship between heat and cold tolerance at both population and individual levels, our design also permitted testing for the first time whether the recently reported reproductive trade-off between these traits (Schou et al., 2022) has a thermoregulatory basis. Japanese quail were raised in cold snap (10°C) or heatwave-like (30°C) temperature conditions from hatching until reproductive maturity, after which half of each treatment group were transferred to common garden conditions at intermediate temperature (20°C) to test whether any temperature-dependent phenotypes stemmed from developmental plasticity or phenotypic flexibility. Cold and heat tolerance was measured halfway to asymptotic size during the exponential growth phase, around the start of reproductive maturity and again well into adulthood. We predicted that birds growing up under warm conditions would cope better in high air temperatures, evaporating water at a higher rate and having a higher heat tolerance limit, compared with birds from cold conditions. By contrast, we predicted that cold rearing would increase thermogenic capacity and improve the cold tolerance limit relative to warm rearing. If these changes were caused by developmental plasticity of the thermoregulatory phenotype and thus are permanently programmed, we predicted that trait values of the common-garden birds would reflect rearing, but not housing conditions (i.e. remain unchanged in common garden conditions) (Fig. 1A,B). However, if thermoregulatory responses reflected phenotypic flexibility as a result of reversible plasticity, we predicted that trait values in the common garden birds would converge (Fig. 1A,B).

## MATERIALS AND METHODS

### Species and husbandry

Japanese quail (*Coturnix japonica* Temminck and Schlegel 1848) eggs (*n*=85) were purchased from a commercial breeder (Sigvard Månsgård, Åstorp, Sweden) and placed in an automatic turner that turned the eggs for 5 min every 1 h at room temperature (18–20°C) for 1–7 days. Then, the eggs were put into one of two Brinsea Ova Easy 190 incubators (6–8 eggs per day; Brinsea, Weston-super-Mare, UK) at 37.45±0.358°C or 37.55±0.205°C (mean±s.d.) and 50% relative humidity (automatically regulated using a Brinsea Ova-Easy Advance Humidity Pump). Eggs were moved from incubation trays to a hatching tray inside the incubator at day 16 of incubation and were checked for hatching twice daily from day 17. Fifty-five eggs of 85 hatched after an incubation period of 19 days (mean±s.d.: 19.27±0.73 days; range: 18–22 days).

When completely dry (1 to 12 h after hatching), the chicks were banded with uniquely numbered leg bands and randomly allocated to either a cold snap-like (10°C; 9.70±0.31°C; *n*=25; Table 1) or a heatwave-like housing regime (30°C; 30.02±0.82°C; *n*=24; Table 1) in line with previous research (Correia et al., 2025; Persson et al., 2024). The birds remained in these thermal environments until 9 weeks old. Then, half of the of the birds (Cold: *n*_female_=6, *n*_male_=6, henceforth Cold–mild; Warm: *n*_female_=6, *n*_male_=5, henceforth Warm–mild) were transferred to a common garden conditions at 20°C (20.01±0.35°C), whereas the other half remained in their original temperature treatments (Cold: *n*_female_=5, *n*_male_=6; Warm: *n*_female_=7, *n*_male_=4) until the end of the experiment (Fig. 1C). Birds reared in warm conditions were often observed panting and displaying thermoregulatory behaviours, such as leg stretching, whereas no such behaviours were observed in the other temperature treatments. The birds were housed in open pens (310×120×60 cm) with food [25.5% protein turkey starter until 3 weeks (Kalkonfoder Start) and 22.5% protein turkey grower from 3 weeks onwards (Kalkonfoder Tillväxt, Lantmännen, Stockholm Sweden)], water, sand and seashells available *ad libitum*. Mealworms, or lettuce and shredded carrots, were provided as diet supplements daily on alternate days. A heat lamp (35–39°C), compensating for the absence of a brooding female, was available until 2 weeks of age (first week: interval during daytime, 110 min on, 10 min off; second week: interval during daytime, 90 min on, 30 min off). The heat lamps were placed so that juveniles had to leave the heat source when foraging or drinking, which is similar to what they experience when alternating between foraging and being brooded by the female in the wild. Body mass (±0.1 g) and wing length (±0.5 mm) were measured once weekly and at 1, 2, 3, 8 and 12 weeks of age, respectively. Analyses and results pertaining to morphological data are shown in Supplementary Materials and Methods. Two days before each cold tolerance measurements (see below), blood samples (<1% of blood volume) were collected from the brachial vein for another study.

**Table 1.** The number of Japanese quail used for investigating responses of rearing in either warm (30°C) or cold (10°C) conditions until reproductive maturity after which half of each treatment group were transferred to common garden conditions (Cold–mild and Warm–mild; 20°C)

Age (weeks)	Total no. birds	Treatment sample size				No. females/males	Sample size in analyses				
		Warm	Cold	Warm–mild	Cold–mild		Body mass	Wing length	Daytime body temp.	Cold tolerance	Heat tolerance
1	49	24	25	–	–	27 | 22	49	49	–	–	–
2	49	24	25	–	–	27 | 22	49	49	–	–	–
3	49	24	25	–	–	27 | 22	49	49	–	47	–
4	48	23	25	–	–	26 | 22	48	–	–	–	41
5	48	23	25	–	–	26 | 22	48	–	–	–	–
6	48	23	25	–	–	26 | 22	48	–	48	–	–
7	47	23	24	–	–	25 | 22	47	–	–	–	–
8	47	23	24	–	–	25 | 22	47	47	–	36	–
9	45	22	23	–	–	24 | 21	45	–	–	–	41
10	44	11	10	11	12	24 | 20	44	–	–	–	–
11	44	11	10	11	12	24 | 20	44	–	42	–	–
12	44	11	10	11	12	24 | 20	44	43	–	44	–
13	44	11	10	11	12	24 | 20	–	–	–	–	43

Except for some experiments and measurements where birds were excluded for reasons stated in the main text, the same birds were used at all ages and at all responses.

Ethical approval was granted by the Malmö/Lund Animal Ethics Committee (acting under the authority of the Swedish Board of Agriculture; permit no. 9426-19).

### Measurement of body temperature

When the birds were 13 to 16 days old, we implanted a sterile temperature-sensitive passive integrated transponder (<0.5% of body weight; LifeChip BioTherm, Destron Fearing, South St Paul, MN, USA) into the intraperitoneal cavity following previously described procedures (Persson et al., 2024). This permitted non-contact measurement of body temperature during cold and heat tolerance measurements. Body temperature was also measured in the holding pens between experimental periods (at 5–7 and 10–12 weeks). Since there were, at most, minor differences between treatment groups, these data are not considered further below (see Supplementary Materials and Methods for details; Fig. S3, Table S2).

### Respirometry

Respirometry measurements to derive cold and heat tolerance were performed during daytime between 29 March and 20 June 2022. Representative raw data for these experiments are presented in Fig. S1. We defined maximum thermogenic performance (summit metabolic rate) as the highest metabolic rate reached during sliding cold exposure and the cold tolerance limit as the temperature at which summit metabolic rate was attained. These measurements were taken at 3, 8 and 12 weeks (Fig. 1C; Table 1). A 13 liter glass respirometry chamber contained inside a climate test chamber (Weiss Umwelttechnik C180, Reiskirchen, Germany) was ventilated with a dry gas mixture of 79% helium and 21% oxygen (‘helox’; 4.44±0.03 l min^−1^, measured using an Alicat 20SLPM mass flow meter; Alicat Scientific Inc., Tucson, AZ, USA) from which we subsampled 128.27±1.25 ml min^−1^ (SS4 pump, Sable Systems, Las Vegas, NV, USA) to measure water vapour density and oxygen consumption (using RH300 and FC-10 analysers, respectively; Sable Systems). Water vapour and carbon dioxide were removed from the airstream before measuring oxygen consumption, using drierite and ascarite, respectively. After thermal acclimation (15 min; 3 weeks at 5°C; 8 and 12 weeks at −5°C), chamber temperature was acutely decreased to −5°C (3 weeks) or −10°C (8 and 12 weeks) after which temperature decreased continuously by 20°C per hour. An experiment ended (a) when oxygen consumption started to decrease for a decrease in chamber temperature; or (b) when there was no change in oxygen consumption for at least 20 min whilst air temperature decreased (Fig. S1A). Immediately after, the bird was placed in a small cage (52×32×30 cm) with access to water and a heat lamp where they remained until being transferred back to their pen 20–40 min later.

We defined the heat tolerance limit as the temperature above which the bird could no longer increase its evaporative water loss in response to heating and derived maximal evaporative capacity based on evaporative water loss recorded at this temperature. Heat tolerance measurements were taken 4–9 days after the cold tolerance assay starting at 4, 9 and 13 weeks (Fig. 1C; Table 1). The same instruments were used for both assays. For heat tolerance, dry (drierite) atmospheric air (10.06±0.01 l min^−1^) was pushed through the 13 liter chambers, of which 403.33±0.28 ml min^−1^ was subsampled for analyses of water vapor density and oxygen. A metal grid platform was placed inside the respirometry chamber over a 1.5–2 cm layer of mineral oil, to remove any influence of evaporation from faeces on evaporative water loss. After acclimation in 30°C (for 30–60 min until stable gas traces), chamber temperature was acutely raised to 40°C, after which it was increased in 2°C increments as soon as gas traces had remained stable for at least 5 min (Noakes et al., 2016; Talbot et al., 2018). Experiments ended (a) when oxygen consumption or evaporative water loss decreased sharply when chamber temperature increased; (b) if body temperature increased >45°C; or (c) if birds showed signs of stress or loss of coordination (McKechnie et al., 2016; Whitfield et al., 2015; Fig. S1B). Immediately after, ethanol (70%) was sprayed onto the ventral plumage and skin and the birds were placed in front of a fan (Whitfield et al., 2015) in the small cage described above, with water *ad libitum* until being transferred to the holding pen 20–40 min later. Birds recovered body temperature within minutes of being placed in front of the fan (E.P., pers. obs.).

During all tolerance assays, we measured air temperature inside the chambers at floor and ceiling level using copper-constantan thermocouples (36-gauge type T) attached to a TC-2000 thermocouple box (Sable Systems) and manually adjusted the climate chamber settings to keep bird temperature at target. All respirometry sessions started and ended with a recording of reference air (i.e. baseline).

### Respirometry data

Respirometry data were extracted using ExpeData (v. 1.9.27; Sable Systems). In cold tolerance measurements, we extracted mean values of the 10 min preceding the endpoint (Swanson et al., 1996). In heat tolerance measurements, we extracted data corresponding to the most stable 2 min of oxygen consumption at the heat tolerance limit. Oxygen consumption (ml min^−1^) was calculated using eqn 11.1 in Lighton (2008) and was converted to metabolic heat production (W) by assuming an energy equivalence of 20 J per 1 ml of O_2_ (Kleiber, 1961). Evaporative water loss (g h^−1^) was calculated using eqn 11.9 in Lighton (2008) and converted to evaporative heat loss (W) by assuming that evaporation of 1 ml water requires 2406 J (Wallace and Hobbs, 2006). Evaporative cooling capacity was calculated as the ratio between evaporative heat loss and metabolic heat production (which makes evaporative cooling capacity dimensionless; Lasiewski et al., 1966). This metric strongly predicted the heat tolerance limit at all ages (Fig. S2; Table S1).

### Final sample sizes

Of the 55 birds that hatched, six died within the first week of life, and another 5 birds died of natural causes during the experiment. Two birds were never considered for the metabolic measurements since additional measurements could not be fitted within the birds' photophase. One bird was removed prematurely from the heat tolerance measurements at 4 weeks owing to stress and was not considered further in the analyses. At 12 weeks, one bird was removed from wing length analyses because its primary feathers were damaged. Final sample sizes for each measurement period and trait are presented in Table 1.

### Statistics

All statistical analyses were performed using R v. 4.3.0 (r-project.org). Linear mixed models (lmer) in *lme4* (https://CRAN.R-project.org/package=lme4; Bates et al., 2015) were used to analyse whether thermal and metabolic responses differed between the cold and warm treatment groups. Metabolic heat production, summit metabolic rate, evaporative water loss, evaporative cooling capacity, heat and cold tolerance limits and body temperature were used as response variables, with Treatment, Age and Treatment×Age as factors. Body mass (mean centred by treatment and age as we expected that treatment would affect mass confounding the body mass variable with the treatment variable) was used as a continuous covariate. Bird ID was used as a random intercept to account for repeated measurements. The effects of the transfer to common garden were analysed at the final metabolic measurement period using ANOVA (anova function in stats package), comparing Warm–mild birds against Warm birds, and Cold–mild birds against Cold birds. All dependent variables but tolerance limits and body temperature were log-transformed in all analyses above to meet parametric assumptions.

The phenotypic association between evaporative cooling capacity and heat-producing capacity (i.e. summit metabolic rate; mean centred by treatment and age), potentially indicative of a trade-off between heat and cold tolerance, was addressed in Cold and Warm birds. Firstly, we fitted using ANOVA (anova function) models to test for differences in slopes between treatments (i.e. Treatment×Summit metabolic rate interaction), separately for each age interval (i.e. 3–4, 8–9 and 12–13 weeks of age). Since responses were uniform across Cold and Warm birds (3–4 weeks: d.f.=1, *F*=0.81, *P*=0.37; 8–9 weeks: d.f.=1, *F*=0.07, *P*=0.77; 12–13 weeks: d.f.=1, *F*=3.40, *P*=0.08), we proceeded using Model II regression (lmodel2 function in the *lmodel2* package; https://CRAN.R-project.org/package=lmodel2) to model the relationship between evaporative cooling capacity and summit metabolism, fitting separate regressions to each age interval.

*P*-values from linear mixed models were assessed using likelihood ratio tests. Non-significant interactions were removed from the models, but all main effects were kept. To interpret and compare significant interactions and effects a *post hoc* test (pairs in *emmeans*; https://CRAN.R-project.org/package=emmeans) were used and estimates were back-transformed when applicable. Significant effects from ANOVAs were compared using a Tukey *post hoc* test (glht in the *multcomp* package; https://CRAN.R-project.org/package=multcomp; Hothorn et al., 2008).

## RESULTS

### Cold-reared birds improved cold tolerance

**Fig. 2.**
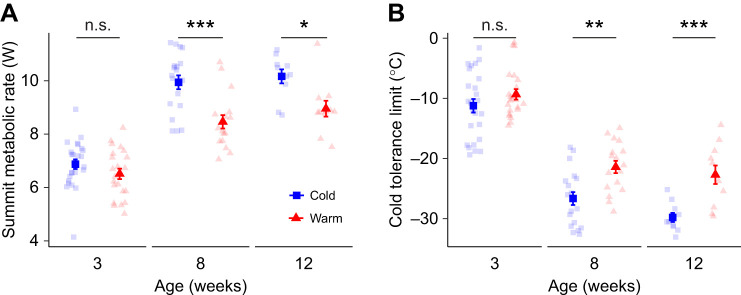
**Maximum thermogenic capacity and cold tolerance limit in temperature-acclimated Japanese quail.** Birds were raised in either Cold (10°C) or Warm (30°C) conditions until 13 weeks of age. Summit metabolic rate (A) and cold tolerance limit (B) were measured at 3, 8 and 12 weeks. Sample sizes per age and temperature are stated in Table 1. Points and error bars show estimated means±s.e. Semi-transparent points show raw data, asterisks represent significance (n.s., *P*>0.05; 0.05≥*P>0.01; 0.01≥**P>0.001; ***P≤0.001).

Birds that grew up under cold snap-like conditions had significantly higher summit metabolic rate at 8 (16.9%) and 12 (12.4%) weeks of age compared with birds from warm conditions, but this effect was absent at 3 weeks (Treatment×Age interaction: *P*=0.060; Fig. 2A; Table 2). When the interaction was removed from the model, Cold birds had 10.4% higher summit metabolic rate than Warm birds (Fig. 2A; Table 2). In addition, summit metabolic rate increased with age and was 37.6% higher at 8 weeks and 43.8% higher at 12 weeks than at 3 weeks of age (Fig. 2A; Table 2). The cold tolerance limit was affected by the interaction between treatment and age. Specifically, Cold birds reached significantly lower temperature than Warm birds at 8 (by 5.1°C) and 12 (by 6.9°C) weeks of age, but there was no significant difference at 3 weeks (Fig. 2B; Table 2). Body temperature at the cold tolerance limit was not affected by the interaction between treatment and age nor by treatment or age alone (Fig. S4A; Table 2).

**Table 2.** Parameter estimates explaining the effects of growing up in either Cold (10°C) or Warm (30°C) temperature conditions in Japanese quail

	Model	Treatment	Estimate±s.e.	LR	d.f.	*P*	σ_ID_|σ_total_
**Cold tolerance**							
Summit metabolic rate*	Treatment×Age			5.62	2	0.0601	
	3 weeks	Cold [A]	6.79±0.157				
		Warm [A]	6.44±0.160				
	8 weeks	Cold [A]	9.83±0.260				
		Warm [B]	8.41±0.236				
	12 weeks	Cold [A]	10.10±0.367				
		Warm [B]	8.99±0.311				
	Treatment			12.51	1	*0.0004*	
		Cold [A]	8.73±0.161				
		Warm [B]	7.91±0.152				
	Age			121.74	2	*<0.0001*	
	3 weeks [A]		6.62±0.114				
	8 weeks [B]		9.11±0.178				
	12 weeks [B]		9.52±0.234				
	Body mass		1.002±1.000	9.64	1	*0.0019*	
	Bird ID (random)			3.25	1	0.0717	0.00|0.01
Cold tolerance limit (°C)	Treatment×Age			6.69	2	*0.0353*	
	3 weeks	Cold [A]	−11.16±0.913				
		Warm [A]	−9.41±0.973				
	8 weeks	Cold [A]	−26.51±1.043				
		Warm [B]	−21.43±1.105				
	12 weeks	Cold [A]	−29.90±1.432				
		Warm [B]	−23.00±1.366				
	Body mass			1.18	1	0.2768	
	Bird ID (random)			2.17	1	0.1408	4.34|20.83
Body temperature (°C)	Treatment			0.35	1	0.5543	
	Age			3.16	2	0.2062	
	Treatment×Age			1.81	2	0.4047	
	Body mass			0.002	1	0.9612	
	Bird ID (random)			8.86	1	*0.0029*	0.62|1.67
**Heat tolerance**							
Metabolic heat production*	Treatment×Age			6.76	2	*0.0341*	
	4 weeks	Cold [A*]	3.20±0.133				
		Warm [A*]	2.89±0.118				
	9 weeks	Cold [A]	3.72±0.151				
		Warm [A]	3.89±0.162				
	13 weeks	Cold [A]	3.67±0.207				
		Warm [A]	3.98±0.216				
	Body mass		1.004±1.001	24.63	1	*<0.0001*	
	Bird ID (random)			8.32	1	*0.0039*	0.01|0.03
Evaporative water loss*	Treatment			6.73	1	*0.0095*	
		Cold [A]	3.54±0.117				
		Warm [B]	4.00±0.135				
	Age			71.26	2	*<0.0001*	
	4 weeks [A]		3.15±0.082				
	9 weeks [B]		4.02±0.104				
	13 weeks [B]		4.17±0.136				
	Treatment×Age			3.25	2	0.1921	
	Body mass			0.14	1	0.7107	
	Bird ID (random)			36.43	1	*<0.0001*	0.02|0.03
Evaporative cooling capacity*	Treatment			3.37	1	0.0663	
	Age			0.94	2	0.6258	
	Treatment×Age			3.27	2	0.1945	
	Body mass		0.996±1.001	14.44	1	*0.0001*	
	Bird ID (random)			36.03	1	*<0.0001*	0.05|0.07
Heat tolerance limit (°C)	Treatment×Age			8.33	2	*0.0155*	
	4 weeks	Cold [A]	43.1±0.453				
		Warm [B]	45.1±0.454				
	9 weeks	Cold [A]	44.1±0.446				
		Warm [A]	44.7±0.460				
	13 weeks	Cold [A]	43.6±0.545				
		Warm [B]	45.5±0.539				
	Body mass		0.975±1.007	12.23	1	*0.0005*	
	Bird ID (random)			38.59	1	*<0.0001*	3.28|4.54
Body temperature (°C)	Treatment×Age			10.97	2	*0.0042*	
	4 weeks	Cold [A]	44.69±0.078				
		Warm [B]	44.45±0.076				
	9 weeks	Cold [A]	44.58±0.076				
		Warm [A]	44.59±0.078				
	13 weeks	Cold [A]	44.45±0.106				
		Warm [A]	44.72±0.102				
	Body mass			0.57	1	0.4492	
	Bird ID (random)			9.62	1	*0.0019*	0.04|0.12

Estimates, test statistics, degrees of freedom, *P*-values and standard deviations (σ) for the random factor from linear mixed models at three different ages, in cold (measured in 79% helium and 21% oxygen) and heat tolerance measurements. Back-transformed model estimates for log-transformed variables (*) are shown in the table. Statistics for main effects are not provided when the interaction was significant. Significant (*P*<0.05) *post hoc* comparisons are shown by different letters within square brackets, asterisks indicate a trend between treatments (*P=*0.081) and *P*<0.05 is indicated in italics.

### Warm-reared birds improved heat tolerance

**Fig. 3.**
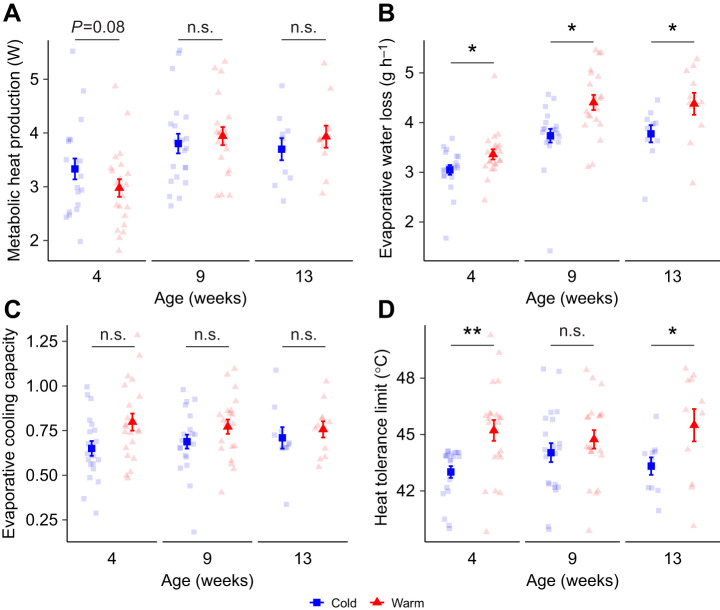
**Thermoregulatory traits at the heat tolerance limit in temperature-acclimated Japanese quail.** (A) Metabolic heat production, (B) evaporative water loss, (C) evaporative cooling capacity and (D) heat tolerance limit. Birds were raised in Cold (10°C) or Warm (30°C) conditions. Sample sizes per age and temperature are stated in Table 1. Points and error bars show estimated means±s.e. Semi-transparent points show raw data, asterisks represent significance (n.s., *P*>0.05; 0.05≥*P>0.01; 0.01≥**P>0.001).

Across ages, warm-acclimated birds had 13.0% higher evaporative water loss at the heat tolerance limit than Cold birds (Fig. 3B; Table 2). In addition, evaporative water loss increased with age, being lower at 4 weeks compared with the two older age groups (Fig. 3B, Table 2). Evaporative cooling capacity was not affected by the interaction between treatment and age, nor by treatment or age alone. However, there was a tendency (*P*=0.066) for Warm birds to have higher evaporative cooling capacity than Cold birds (Fig. 3C; Table 2). Nevertheless, Warm birds had a higher heat tolerance limit compared with Cold birds (at 4 and 13 weeks: 2.0°C and 1.9°C respectively; Fig. 3D, Table 2). There was no significant difference in metabolic heat production at the heat tolerance limit between Warm and Cold birds at any age (Fig. 3A; Table 2). However, metabolic heat production was higher at 9 and 13 weeks of age than at 4 weeks. Specifically, Warm birds had significantly higher metabolic heat production at 9 and 13 weeks of age (9 weeks: 34.6%, *P*<0.0001; 13 weeks: 37.7%, *P*<0.0001) than at 4 weeks, whereas Cold birds had significantly higher metabolic heat production at 9 weeks (16.3%, *P*=0.008) than at 4 weeks of age (Treatment×Age interaction: *P*=0.038). Body temperature at the heat tolerance limit was affected by the interaction between treatment and age (Fig. S4B; Table 2) such that at 3 weeks, Cold birds had 0.24°C higher body temperature than Warm birds at heat exhaustion.

### Effect-reversal in common garden birds

**Fig. 4.**
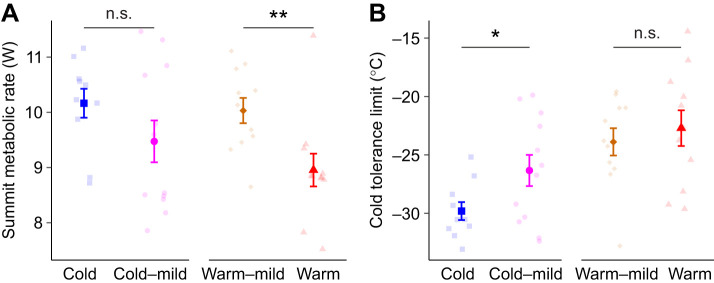
**Effects of transfer to common garden conditions on summit metabolic rate and cold tolerance limits.** Japanese quail were reared in Cold (10°C) or Warm (30°C) temperature until 9 weeks, after which half of the group was transferred to common garden conditions (20°C). (A) Summit metabolic rate and (B) cold tolerance limit at 12 weeks. Sample sizes per age and temperature are stated in Table 1. Points and error bars show estimated means±s.e. Semi-transparent points show raw data, asterisks represent significance (n.s., *P*>0.05; 0.05≥*P>0.01; 0.01≥**P>0.001).

**Fig. 5.**
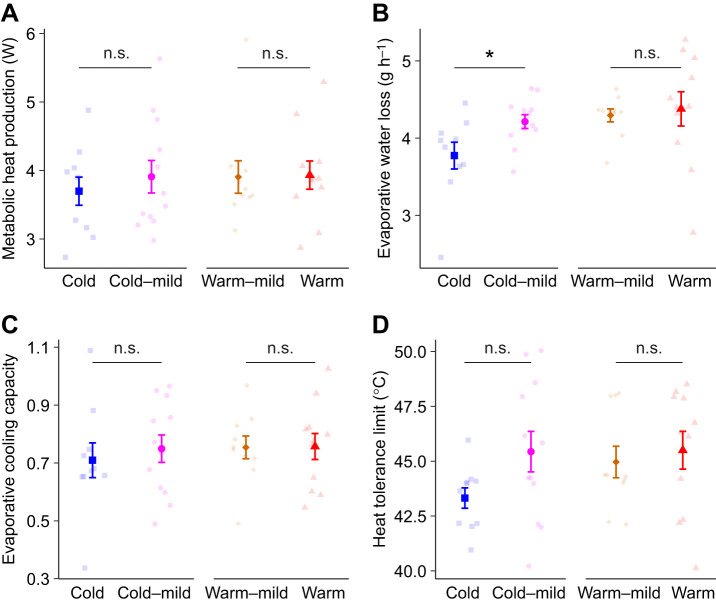
**Effects of transfer to common garden conditions on heat tolerance traits.** Birds were reared in Cold (10°C) or Warm (30°C) until 9 weeks of age, after which they spent 3 weeks at a common temperature (20°C) before being measured again. (A) Metabolic heat production, (B) evaporative water loss, (C) evaporative cooling capacity and (D) heat tolerance limit. Sample sizes per age and temperature are stated in Table 1. Points and error bars show estimated means±s.e. Semi-transparent points show raw data, asterisks represent significance (n.s., *P*>0.05; 0.05≥*P>0.01).

After the transfer to common garden conditions, Warm–mild birds developed 12.4% higher summit metabolic rate than birds that remained in Warm conditions (Fig. 4A; Table 3). There was no significant difference in summit metabolic rate between Cold–mild and Cold birds. However, Cold–mild birds reduced their cold tolerance limit compared with Cold birds (by 3.5°C; Fig. 4B; Table 3). In contrast, the heat tolerance limit did not differ between Warm and Warm–mild birds. Evaporative water loss improved in Cold–mild compared with Cold birds (by 12.6%), with a tendency for an analogous change in the heat tolerance limit (*P*=0.076; Fig. 5A,D; Table 3). There was no difference in these traits between Warm–mild and Warm birds. Body temperature at the tolerance limits did not differ between Cold and Cold–mild birds, nor between Warm and Warm–mild birds (Fig. S5A,B; Table 3).

**Table 3.** Parameter estimates explaining the effects of moving from either cold (10°C) or warm (30°C) conditions to common garden conditions (20°C) in Japanese quail

	Treatment	Model	Estimate±s.e.	*F*	d.f.	*P*
**Cold tolerance – 12 weeks**						
Summit metabolic rate*	Cold∼Cold–mild	Treatment		2.96	1/19	0.1016
		Body mass	1.002±1.001	6.33	1/19	*0.0211*
	Warm∼Warm–mild	Treatment		14.19	1/19	*0.0013*
		Warm [A]	8.91±0.194			
		Warm–mild [B]	10.01±0.218			
		Body mass	1.003±1.001	13.58	1/19	*0.0016*
Cold tolerance limit (°C)	Cold∼Cold–mild	Treatment		5.06	1/19	*0.0365*
		Cold [A]	−29.80±1.140			
		Cold–mild [B]	−26.30±1.104			
		Body mass		3.06	1/19	0.0965
	Warm∼Warm–mild	Treatment		0.46	1/19	0.5067
		Body mass	−0.109±0.047	5.45	1/19	*0.0307*
Body temperature (°C)	Cold∼Cold–mild	Treatment		3.20	1/19	0.0894
		Body mass		0.12	1/19	0.7299
	Warm∼Warm–mild	Treatment		0.11	1/19	0.7388
		Body mass		0.03	1/19	0.8569
**Heat tolerance – 13** **weeks**						
Metabolic heat production*	Cold∼Cold–mild	Treatment		0.38	1/19	0.5447
		Body mass	1.002±1.001	0.53	1/19	0.4753
	Warm∼Warm–mild	Treatment		0.01	1/18	0.9187
		Body mass	1.004±1.002	5.11	1/18	*0.0364*
Evaporative water loss*	Cold∼Cold–mild	Treatment		5.61	1/19	*0.0287*
		Cold [A]	3.73±0.138			
		Cold–mild [B]	4.20±0.142			
		Body mass		3.37	1/19	0.0822
	Warm∼Warm–mild	Treatment		0.01	1/18	0.9174
		Body mass	1.000±1.001	0.71	1/18	0.4103
Evaporative cooling capacity*	Cold∼Cold–mild	Treatment		0.35	1/19	0.5605
		Body mass		0.08	1/19	0.7827
	Warm∼Warm–mild	Treatment		0.00	1/18	0.9962
		Body mass		1.48	1/18	0.2392
Heat tolerance limit (°C)	Cold∼Cold–mild	Treatment		3.51	1/19	0.0764
		Body mass		0.04	1/19	0.8498
	Warm∼Warm–mild	Treatment		0.21	1/18	0.6521
		Body mass		1.12	1/18	0.7350
Body temperature (°C)	Cold∼Cold–mild	Treatment		3.13	1/19	0.0931
		Body mass		0.66	1/19	0.4252
	Warm∼Warm–mild	Treatment		2.38	1/18	0.1404
		Body mass		0.11	1/18	0.7482

Estimates, test statistics, degrees of freedom and *P*-values from ANOVAs at 12 weeks in cold tolerance measurements (measured in 79% helium and 21% oxygen) and at 13 weeks in heat tolerance measurements. Back-transformed model estimates for log-transformed variables (*) are shown in the table. Significant (*P*<0.05) *post hoc* comparisons are shown by different letters within square brackets and *P*<0.05 is indicated in italics.

### The relationship between cooling and warming capacity

**Fig. 6.**
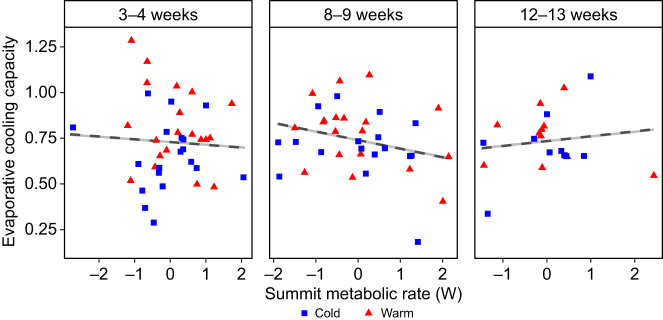
**Relationship between maximum cold and heat tolerance in warm- and cold-acclimated Japanese quail.** Cold tolerance was inferred from maximum thermogenic capacity (summit metabolic rate) during sliding cold exposure and heat tolerance was inferred from the maximum evaporative cooling capacity during heat exposure. Dashed lines represent major axis regression slopes and solid lines represent ordinary least squares slopes (all *P>*0.1). Sample sizes per age and temperature are stated in Table 1 and statistics are presented in Table 4.

There was no evidence that cold or warm acclimation came at the expense of reduced heat or cold tolerance, respectively (Fig. 6). This was reflected in the absence of any significant relationship between evaporative cooling capacity and thermogenic capacity (i.e. summit metabolic rate) at any age. Both the ordinary least square and major axis slopes overlapped zero, and the models explained very little variation (all *R*^2^<0.08; all *P*>0.01; Table 4). Given the shallow slopes and weak correlations, we did not interpret the standardised major axis (SMA) slopes.

**Table 4.** Parameter estimates explaining interaction between treatment and summit metabolic rate and the relation between evaporative cooling capacity and summit metabolic rate at three different ages in Japanese quail

Regression model	*R* ^2^	*P*	Method	Intercept	97.5% CI_Intercept_	Slope	97.5% CI_Slope_
3–4 weeks	0.0035	0.7176	Ordinary least squares	0.7292	0.6590–0.7994	–0.0144	–0.0941–0.0654
			Major axis	0.7292	0.7272–0.7312	–0.0153	–0.1004–0.0697
8–9 weeks	0.0732	0.1105	Ordinary least squares	0.7398	0.6794–0.8003	–0.0460	–0.1030–0.0110
			Major axis	0.7398	0.7398–0.7398	–0.0472	–0.1060–0.0113
12–13 weeks	0.0187	0.5547	Ordinary least squares	0.7343	0.6565–0.8121	0.0257	–0.0637–0.1151
			Major axis	0.7343	0.7343–0.7343	0.0266	–0.0662–0.1199

Estimates, test statistics, degrees of freedom and *P*-values from standardised major axis regressions.

## DISCUSSION

In line with our predictions, we found that Japanese quail growing up under cold snap-like conditions were more thermogenic and had improved cold tolerance limits compared with birds raised in the warmth. Growing up under heatwave-like conditions equipped birds with higher evaporative water loss and improved heat tolerance limits. While heat tolerance limits are typically far beyond temperatures birds are likely to experience over sustained periods in nature, it is plausible that individuals tolerating higher, or lower, temperatures can endure submaximal thermal stress with a lower somatic cost (cf. Ørsted et al., 2022). This could allow the birds to maintain foraging effort, or exploit thermally challenging microhabitats, before having to actively engage in heat loss or heat gain processes, thus reducing missed opportunity costs of temperature variability (Cunningham et al., 2021).

Differences between birds raised in cold snap and heatwave conditions were largely reversed once the thermal stressor was removed and the birds had spent some weeks in common garden conditions. Since plasticity is assumed to be costly and would be predicted to be lost in stable environments (Morgan et al., 2022), this might suggest that costs of phenotypic plasticity are less pronounced in birds compared with some ectotherm models (Morgan et al., 2022). The timeline of reversal differed between thermoregulatory traits (see Figs 4,5, Fig. S5), which might influence the capacity to deal with thermally labile environments. Moreover, the different timelines between trait reversals highlight the sometimes complex relationships between thermoregulatory traits and temperature tolerance limits (cf. Milbergue et al., 2018) and support recent work of the latter (Rego et al., 2025). Yet, previous research shows that birds can quickly acclimate both muscle ultrastructure and thermoregulatory performance when environmental temperature changes (Petit et al., 2013; Vezina et al., 2020). Furthermore, reversal of temperature-dependent phenotypes aligns with previous endotherm studies showing that postnatal temperature conditions do not permanently program thermoregulation (Liew et al., 2003; Persson et al., 2024). It is likely that adaptive developmental priming requires close mapping of the environmental temperature in juvenile and adult life stages. However, this may not apply for highly mobile animals such as birds, where plasticity instead could ensure matching of the thermoregulatory phenotype to the surrounding thermal environment over short time frames. Alternatively, as the endothermic nature of birds renders them physiologically or functionally homeothermic from an early age (e.g. Andreasson et al., 2016; Engert et al., 2025), any window for developmental plasticity may close beyond this point. In line with this, the poikilothermic embryonic period appears more malleable to developmental plasticity of temperature tolerance in birds (reviewed by Arjona et al., 1988; Piestun et al., 2008).

An outstanding question is whether heat and cold tolerance are subjected to a trade-off, such that an individual can be either more thermogenic or more thermolytic, but not both. Likewise, there has been a recent call for understanding how any such trade-off may differ during development in line with ontogenetic changes in an individual's thermal relations (Schou and Cornwallis, 2024). Existing evidence for a trade-off is available at the level of reproductive investment, where ostriches that maintained a high egg laying rate in the cold reduced investment in the warmth, and vice versa. The physiological basis for this relationship remains unstudied (Schou et al., 2022). While our treatment groups differed expectedly in heat and cold responses, we found no relationship between heating and cooling capacity. Hence, although populations may vary in heat and cold tolerance, we found no evidence for a trade-off in quail, possibly because thermogenesis and thermolysis follow largely distinct physiological pathways. Different pathways are also consistent with the timelines for the reversal of thermoregulatory traits (above), suggesting that there is no single mechanism controlling changes in evaporation (heat tolerance) and summit metabolic rate (cold tolerance). Quail can evaporate water using gular flutter, which is comparatively inexpensive energetically (O'Connor et al., 2017; Talbot et al., 2017). It is therefore possible that a significant relationship between heat and cold tolerance only emerges in species where evaporative cooling is comparatively more energetically demanding, such as passerines, which are phylogenetically constrained to rely on panting (e.g. McKechnie et al., 2016, 2021; Smit et al., 2018). The direction of such a relationship could plausibly range from negative to positive, depending on whether traits that enhance heat tolerance increase basal heat production or instead improve the performance of the musculature used during panting. Moreover, trade-offs between heat- and cold tolerance could be evident in traits not measured in this study. In line with this, we recently showed that cold-reared quail from this study exhibited reduced heat tolerance of mitochondrial function during acute heat exposure (Correia et al., 2025). By extension, such subcellular trade-offs could also help to explain the complex and sometimes opposing relationships between postnatal developmental temperature and telomere attrition or elongation reported across diverse bird species (Eastwood et al., 2022, 2023; Ton et al., 2023). We recommend that these questions are addressed further in a wider range of species across latitudes and biomes.

In contrast to previous work showing no effects of cold acclimation during a mild cold challenge (Persson et al., 2024), our results demonstrate that thermoregulatory traits at cold tolerance limit are affected, even though the cold acclimation was the same in both cases. This suggests that measurements both below and at thermal limits are necessary to capture the full dynamics of postnatal developmental temperature effects. Moreover, thermoregulatory traits mediating cold tolerance increased with age in a treatment-specific manner, such that there were no significant differences in thermogenic capacity or cold tolerance limits in the juveniles (Fig. 2). This may reflect a trade-off between thermoregulation and growth. It is reasonable that the lower absolute amount of thermogenic tissue at halfway to asymptotic size renders the birds less cold tolerant. Moreover, the juvenile plumage is often thinner and less insulating (Nord et al., 2023), which drives up heat loss rate (Nord and Folkow, 2018). When combining these predicaments, juvenile assimilation efficiency may simply be insufficient to maximise growth rate (required to reduce predation risk; e.g. Cheng and Martin, 2012; Martin, 1995) whilst simultaneously increasing investment in thermogenic tissue (e.g. Krijgsveld et al., 2003), explaining the lack of differences between treatments. At and beyond reproductive maturity, cold snap-reared birds displayed the expected increase in thermogenic performance and cold tolerance limit. It is noteworthy that this might not reflect increased mass of heat-producing tissue in cold-exposed birds, since there was no overall difference in body size between the treatments (Fig. S6; Tables S3,S4). Thus, our study supports the conclusion by Milbergue et al. (2018) that while larger muscles may be beneficial for heat production, they are not a strict necessity for improved cold tolerance. Future studies should investigate both the aerobic activity of skeletal muscle in cold-reared birds (cf. Liknes and Swanson, 2011) and any temperature-related differences in plumage insulation (cf. Osváth et al., 2018; Pap et al., 2020).

We have shown that, although postnatal developmental temperature impacts the ontogeny of thermoregulation, these effects appear to be reversible. This suggests that the effects caused by temperature changes during postnatal development reflect phenotypic flexibility and not developmental plasticity. Moreover, and importantly, the scope for acclimating to cold and warm conditions differed. Whereas the difference in cold tolerance limit between treatments was approximately 7°C, the increase in heat tolerance limit in warm-exposed birds was only around 2°C. This was also apparent when comparing the common garden birds with their original thermal groupings: evaporative water loss and the heat tolerance limit was downregulated in birds exposed to cold, but it was not upregulated in birds exposed to warmth. Similar results were recently found at the cellular level, where the thermal sensitivity of blood cell mitochondria were amenable to cold acclimation but insensitive to warm acclimation (Correia et al., 2025). While we can only speculate about the physiological processes mediating differences in acclimation capacity to heat and cold, it is likely that there are hard ceilings for water turnover rate, molecular chaperones protecting cells from heat stress (e.g. heat shock proteins; Dubrez et al., 2020) and other yet-to-be-identified mechanisms that preclude close matching of heat exposure and heat tolerance, increasing endotherm sensitivity to detrimental hyperthermia under climate warming. Even though the absence of a detectable trade-off between heat and cold tolerance suggests that cold-adapted endotherms may not be at elevated risk, we share the concerns raised for ectotherms (Jørgensen et al., 2022; Morgan et al., 2020) that a limited scope for improving heat tolerance is troubling in a warming world. When physiological flexibility is constrained, animals may be forced to rely primarily on behavioural adjustment, which can carry potentially severe downstream fitness costs (cf. Cunningham et al., 2021).

## Supplementary Material

10.1242/jexbio.251867_sup1 Supplementary information
