# Supplementary material for: Postnatal temperature triggers predictable thermoregulatory shifts in birds without a trade-off between heat and cold tolerance
Source: J Exp Biol. 2026 May 13;229(9):jeb251867. doi: 10.1242/jeb.251867 (PMC13245907; doi:10.1242/jeb.251867)
Supplement: Supplementary information [file jexbio-229-251867-s1.pdf]

Supplementary Materials and Methods

Temperature tolerance measurements

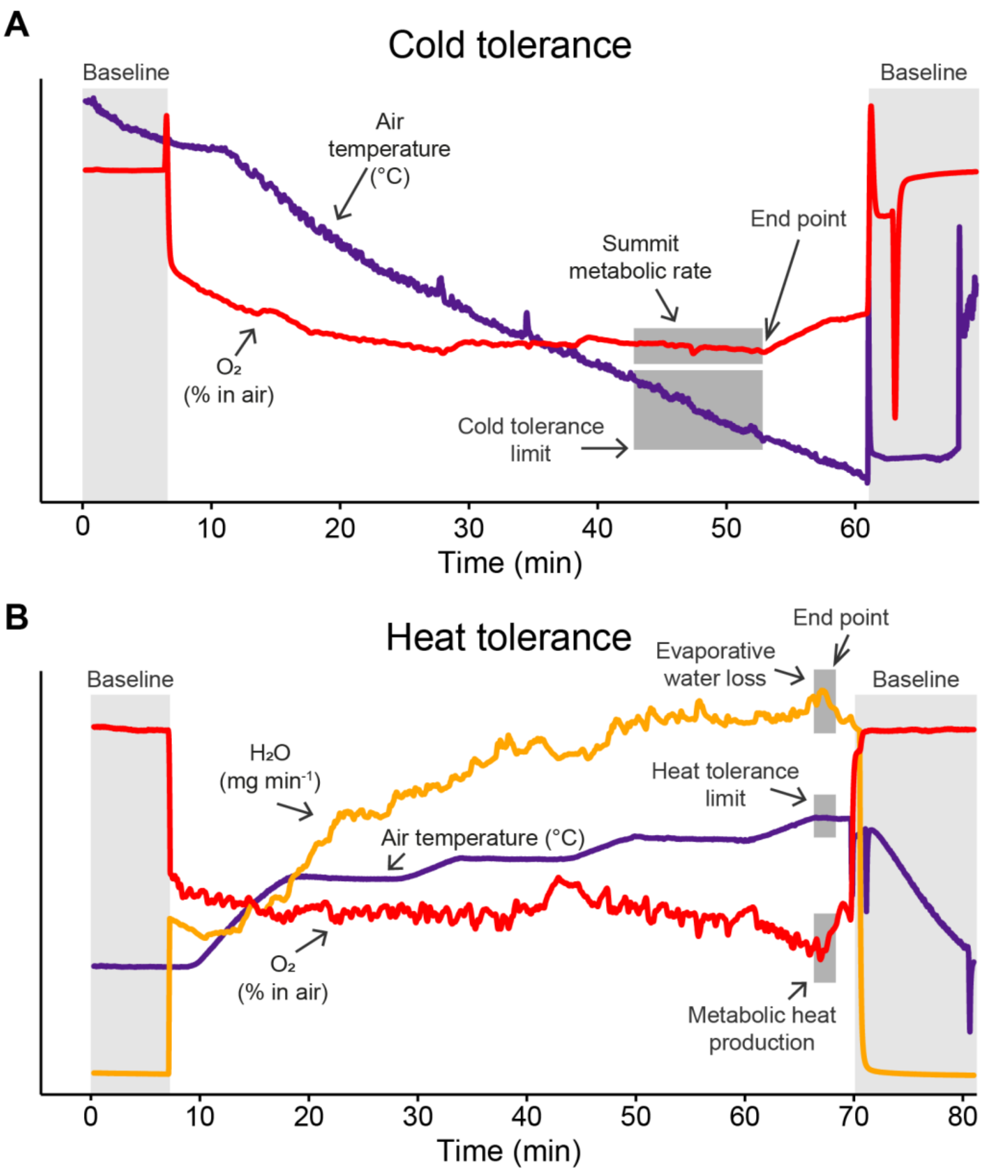

Fig. S1. Representative examples of (A) cold- and (B) heat tolerance measurements. (A) A cold-tolerance experiment was performed in a helium-oxygen gas mixture (79% helium, 21% oxygen). Air temperature (purple line) decreased by 20°C per hour. O_2-curve represents percentage of O_2 in air. The percentage of O_2 in air (red line) decreases with time and decreasing air temperature, reflecting increased thermogenesis. At thermoregulatory failure, O_2 consumption starts decreasing with decreasing air temperature, reflected in increased percentage of O_2 in air. A bird was removed from the experiment at this end point, or when oxygen consumption had remained stable for at least 20 min in decreasing air temperature. Metabolic data in the shaded dark grey area

Journal of Experimental Biology • Supplementary information

(10 min leading up to the end point) were averaged and used to calculate cold tolerance limit and summit metabolic rate. (B) In the heat tolerance measurements, air temperature was acutely increased to 40°C after 30 min acclimation in 30°C (not depicted in the graph), and then in 2°C increments as soon as gas concentration curves had remained stable for at least 5 min. A bird was assumed to have reached its' end point when oxygen consumption or water vapor concentration (H_2O; evaporative water loss; yellow line) started to decrease, if body temperature increased > 45°C, or if birds showed loss of coordination or signs of stress. Averages of the shaded dark grey areas (most stable 2 min before the end point) were used to calculate the heat tolerance limit, and its associated evaporative water loss and metabolic heat production. The shaded light grey areas show baseline measurements.

Journal of Experimental Biology • Supplementary information

Relationship between the heat tolerance limit and evaporative cooling capacity

To investigate if evaporative cooling capacity could be used to predict the heat tolerance limit, we analysed the relationship between evaporative cooling capacity and heat tolerance limit using linear regression (lm() in the stats package) with heat tolerance limit as the dependent variable and evaporative cooling capacity as the independent variable.

Evaporative cooling capacity significantly predicted heat tolerance limit, which increased by 6.97°C at 4 weeks, 10.23°C at 9 weeks and 10.82°C at 13 weeks for each unit increase in the capacity for evaporative cooling (Fig. S2; Table S1).

Table S1. Parameter estimates from linear regressions between heat tolerance limit (i.e., the temperature above which a bird no longer could increase its evaporative water loss) and evaporative cooling capacity (i.e., the ratio between evaporative heat loss and metabolic heat production) in Japanese quail at 3 different ages.

Model	Intercept	Slope	*t*	R^2	d.f.	*P*
**Heat tolerance limit (°C) vs. Evaporative cooling capacity**						
4 weeks	39.08 ± 1.005	6.97 ± 1.329	5.25	0.40	1,39	*<0.0001*
9 weeks	36.93 ± 0.850	10.23 ± 1.132	9.03	0.67	1,39	*<0.0001*
13 weeks	36.81 ± 1.570	10.82 ± 2.070	5.23	0.39	1,41	*<0.0001*

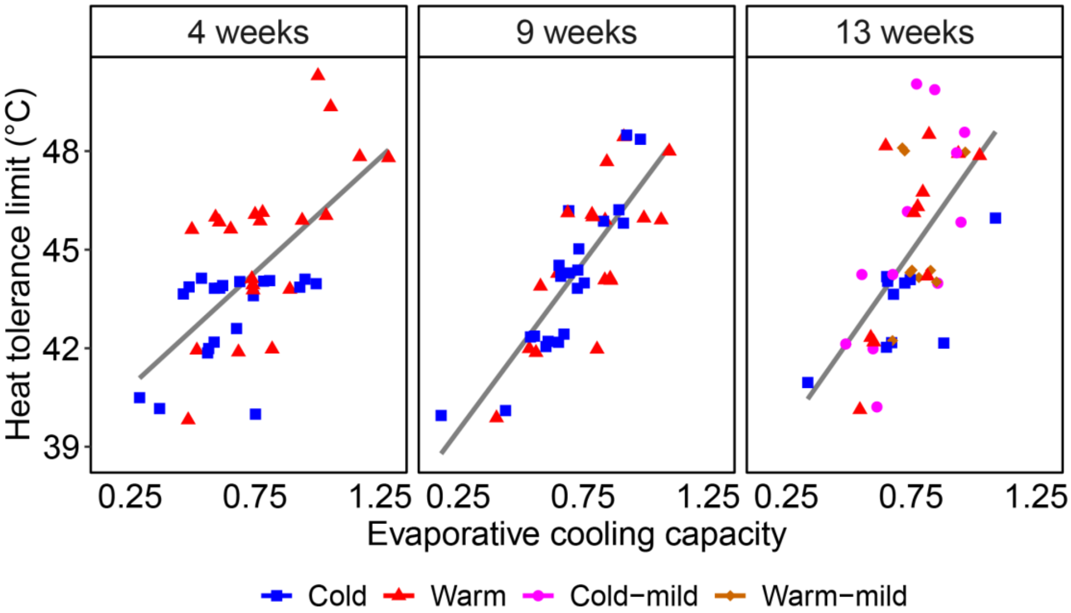

Fig. S2. Heat tolerance limit as a function of maximum evaporative cooling capacity at 4, 9, and 13 weeks of age in Japanese quail. The birds were raised in either Warm (30°C) or Cold (10°C) conditions until 9 weeks of age, after which half of each group was transferred to common-garden conditions (Cold-mild, Warm-mild; 20°C). The other half remained in their original temperature treatments. Grey line shows regression line for respective age. Statistics are presented in Table S1.

Journal of Experimental Biology • Supplementary information

Body temperature

Body temperature was measured using a passive integrated transponder implanted into the intraperitoneal cavity. This allowed readings of body temperature during tolerance measurements as well as daytime measurements in the holding pens. Daytime body temperature (08:00 – 20:00 GMT+2, i.e., 1 h after and before lights off) was measured twice, in-between experimental periods: firstly when the birds were 5-7 weeks old (mean ± s.e.m.: 43.7 ± 4.6 d), and secondly when the birds were 10-12 weeks old (77.2 ± 4.5 d). During these periods, birds were not handled and were left undisturbed apart from daily maintenance. Twelve to 2036 observations were collected per individual at each sampling point (mean: 489 ± 323 observations). Daytime body temperature was analysed using linear mixed models with treatment as a factor, mean centred body mass (by treatment, age and sex) as a covariate, and bird ID as random intercept. Separate models were fitted for the 5-7-week and 10-12-week periods. In the latter case, we compared Cold birds with Warm birds, Warm-mild birds with Warm birds and Cold-mild birds with Cold birds.

Daytime body temperature was higher in Warm compared to Cold birds (by 0.2 ± 0.09°C) in the first measurement period (i.e., at 5 to 7 weeks old; $P = 0.010$; Fig. S3; Table S2), but this difference had disappeared by the second measurement period (at 10 to 12 weeks old). Nor was there a difference in daytime body temperature between the Warm-mild and Warm birds, and Cold-mild and Cold birds, respectively, at this age (Fig. S3; Table S2).

Table S2. Parameter estimates explaining the effects on daytime body temperature of growing up in either Warm (30°C) or Cold (10°C) temperature conditions in Japanese quail, after which half of the birds were transferred to common garden (Cold-mild, Warm-mild; 20°C) and the half remained in their origin treatment. Estimates, test statistics, degrees of freedom, *P*-values and standard deviations (σ) for the random factor from linear mixed models at 5-7 and 10-12 weeks on daytime body temperature. Different letters within brackets represent significant ($P < 0.05$) post hoc comparisons.

Model	Estimate ± s.e.	LR	d.f.	*P*	$\sigma_{ID} \| \sigma_{total}$
**Daytime body temperature**					
5-7 weeks					
Treatment		6.57	1	*0.0104*	
Cold [A]	41.97 ± 0.059				
Warm [B]	42.19 ± 0.061				
Body mass	-0.005 ± 0.002	6.24	1	*0.0125*	
Bird ID (random)		15361.00	1	*<0.0001*	0.06|0.15
10-12 weeks Cold~Warm					
Treatment		0.60	1	0.4347	
Body mass	0.005 ± 0.002	8.71	1	*0.0032*	
Bird ID (random)		4457.30	1	*<0.0001*	0.06|0.31
10-12 weeks Cold~Cold-mild					
Treatment		1.56	1	0.2113	
Body mass		0.05	1	0.8259	
Bird ID (random)		10493.00	1	*<0.0001*	0.07|0.34
10-12 weeks Warm~Warm-mild					
Treatment		3.24	1	0.0717	
Body mass		3.03	1	0.0818	
Bird ID (random)		9768.60	1	*<0.0001*	0.09|0.40

Journal of Experimental Biology • Supplementary information

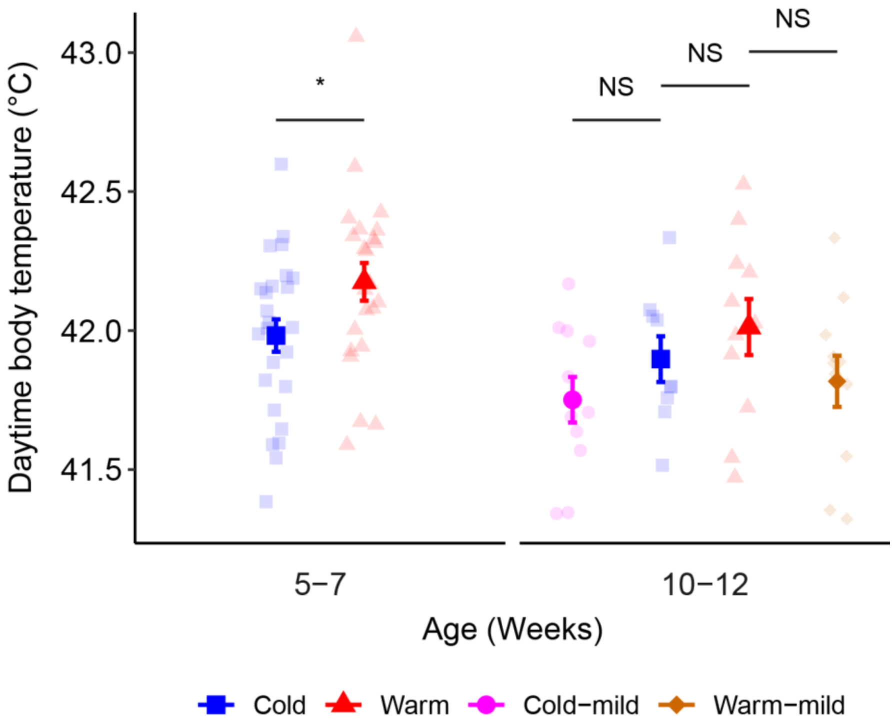

Fig. S3. Body temperature at daytime between tolerance measurements periods in all treatments (mean ± s.e.). Japanese quail were raised in either Warm (30°C) or Cold (10°C) conditions until 9 weeks of age. Then, half of each rearing temperature group were moved to common garden (Cold-mild, Warm-mild; 20°C) and the other half remained in their original rearing temperature treatment (but were moved between cages). Semi-transparent points show raw data; asterisks represent significance levels (NS: $P > 0.05$; *: $0.05 \geq P > 0.01$; **: $0.01 \geq P > 0.001$; ***: $P \leq 0.001$). Sample sizes per age and temperature are stated in Table 1.

Journal of Experimental Biology • Supplementary information

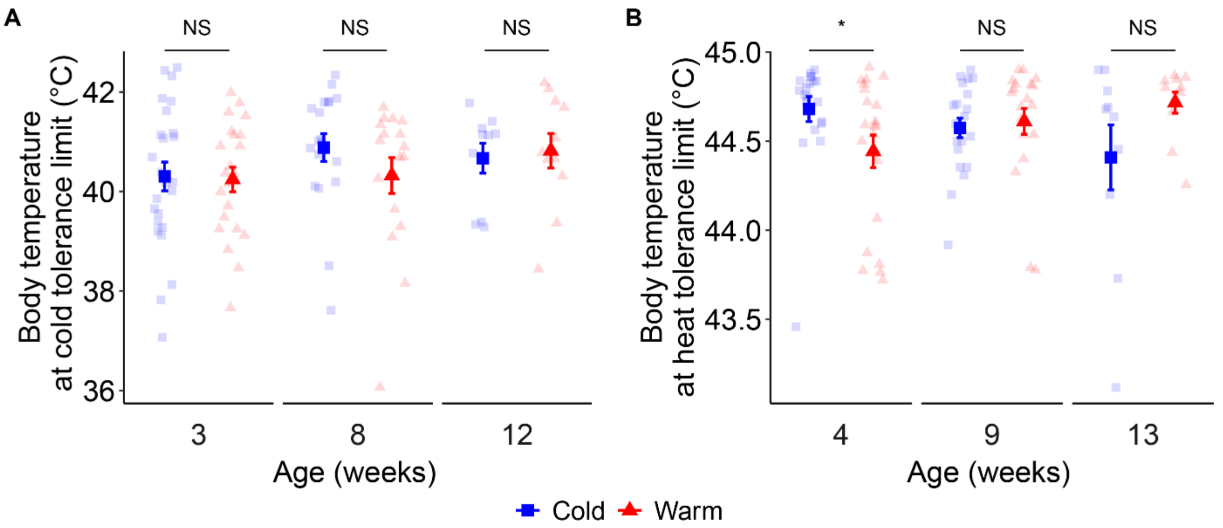

Fig. S4. Body temperature at (A) cold- and (B) heat tolerance limit. Cold tolerance was measured at 3, 8, and 12 weeks of age in 79% helium and 21% oxygen. Heat tolerance was measured at 4, 9 and 13 weeks of age. Japanese quail were raised in either Warm (30°C) or Cold (10°C) conditions until 9 weeks of age. Then, half of the quail were transferred to common garden (Cold-mild, Warm-mild; 20°C) and half remained in their origin temperature treatment. Semi-transparent points show raw data. Sample sizes per age and temperature are stated in Table 1, asterisks represent significance (NS: $P > 0.05$; *: $0.05 \geq P > 0.01$; **: $0.01 \geq P > 0.001$; ***: $P \leq 0.001$).

Journal of Experimental Biology • Supplementary information

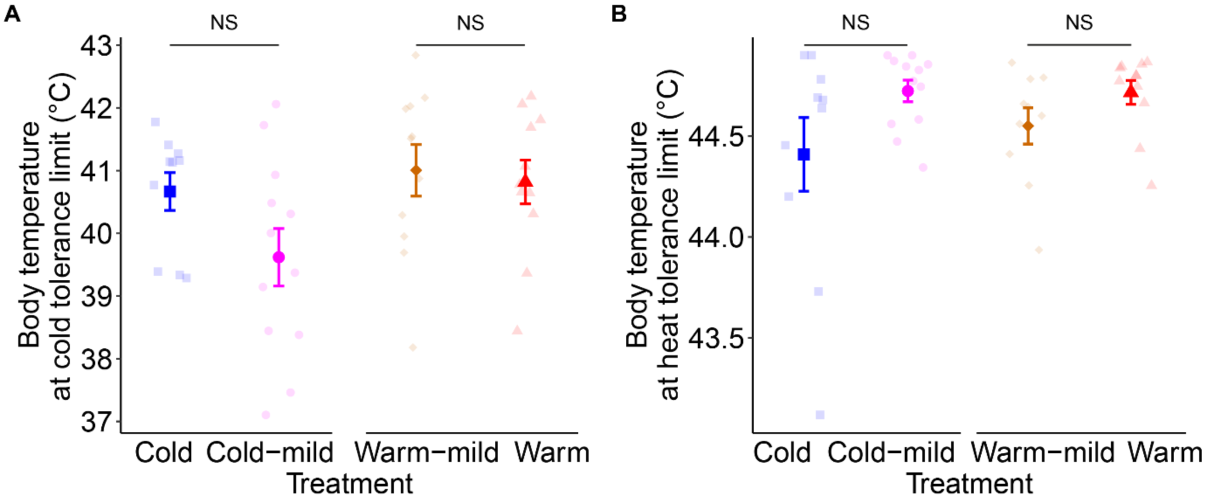

Fig. S5. Effects on body temperature at (A) cold- and (B) heat tolerance limit of transferring to common garden conditions (20°C) in Japanese quail after 9 weeks acclimation to Cold (10°C) or Warm (30°C) conditions. Cold tolerance was in 79% helium and 21% oxygen. Semi-transparent points show raw data. Sample sizes per age and temperature are stated in Table 1, asterisks represent significance (NS: $P > 0.05$; *: $0.05 \geq P > 0.01$; **: $0.01 \geq P > 0.001$; ***: $P \leq 0.001$).

Body mass and wing length

Body mass and wing length were measured to investigate any effects of the developmental temperature treatment on somatic growth and size. To test if body mass and wing length differed between treatment groups and sex at reproductive maturity (9 and 8 weeks, respectively), and at 12 weeks of age, we used ANOVAs (anova() function in stats package) with treatment, sex, and treatment × sex as factors. To test if there was an effect of transfer to common garden conditions, Cold-mild and Warm-mild birds were tested against their origin treatment.

Rearing at cold or warm temperatures affected body mass at 9 weeks in a sex-specific manner, where sexual size dimorphism was less pronounced in Warm compared to Cold birds (Fig. S6A; Table S3). Specifically, Cold males were 22% lighter than Cold females, but Warm males only 11% lighter than Warm females (Fig. S6A; Table S3). In addition, Warm males were lighter than Cold males (P = 0.029), while Warm females only tended to be lighter than Cold females (P = 0.080). At 12 weeks, females weighed more than males across groups (Cold vs. Warm, Cold vs. Cold-mild and Warm vs. Warm-mild), but there was no effect of treatment on body mass (Table S3). At 8 weeks of age (i.e., at asymptotic body mass) there was no effect of treatment on wing length, but males had significantly shorter wings than females (by 2%; Table S4). However, at 12 weeks, Warm birds had 4% longer wings than Cold birds and, again, females had longer wings than males (by 4%; Fig. S6B; Table S4). Wing length did not differ between Cold and Cold-mild birds at 12 weeks.

Table S3. Parameter estimates explaining the effects on body mass of rearing in either Warm (30°C) or Cold (10°C) conditions until 13 weeks. After 9 weeks, half of each treatment group were transferred to common garden (Cold-mild, Warm-mild; 20°C). Estimates, test statistics, degrees of freedom and *P*-values from ANOVAs on body mass. Significant (P < 0.05) post hoc comparisons are shown by different letters within brackets.

**Model**	**Estimate ± s.e.**	***F***	**d.f.**	***P***
**Cold~Warm**				
9 weeks				
Treatment:Sex		8.27	1,41	*0.0064*
Cold				
Female [A]	299.2 ± 8.10			
Male [B]	223.2 ± 11.21			
Warm				
Female [A]	279.5 ± 11.00			
Male [B]	250.0 ± 16.16			
12 weeks				
Treatment		0.99	1,19	0.3323
Sex		19.90	1,19	*0.0003*
Female [A]	287.0 ± 8.04			
Male [B]	232.3 ± 12.28			
Treatment:Sex		2.51	1,17	0.1309
**Cold~Cold-mild**				
Treatment		1.39	1,20	0.2521
Sex		31.01	1,20	*<0.0001*
Female [A]	308.7 ± 10.09			
Male [B]	229.2 ± 14.27			
Treatment:Sex		0.21	1,18	0.6538
**Warm~Warm-mild**				
Treatment		0.46	1,20	0.5038
Sex		15.09	1,20	*0.0009*
Female [A]	292.9 ± 6.71			
Male [B]	252.1 ± 10.45			
Treatment:Sex		0.47	1,18	0.5025

Journal of Experimental Biology • Supplementary information

Table S4. Parameter estimates explaining the effects on wing length of rearing in either Warm (30°C) or Cold (10°C) conditions until 13 weeks. After 9 weeks, half of each treatment group were transferred to common garden (Cold-mild, Warm-mild; 20°C). Estimates, test statistics, degrees of freedom and *P*-values from ANOVAs on wing length. Significant (*P* < 0.05) post hoc comparisons are shown by different letters within brackets.

Model	Estimate ± s.e.	*F*	d.f.	*P*
**Cold~Warm**				
8 weeks				
Treatment		2.38	1,45	0.1298
Sex		5.98	1,45	*0.0184*
Female [A]	124.8 ± 0.68			
Male [B]	122.3 ± 0.99			
Treatment:Sex		2.49	1,43	0.1222
12 weeks				
Treatment		8.47	1,19	*0.0090*
Cold [A]	120.2 ± 1.14			
Warm [B]	124.8 ± 1.16			
Sex		12.39	1,19	*0.0023*
Female [A]	124.8 ± 0.97			
Male [B]	119.6 ± 1.48			
Treatment:Sex		0.05	1,17	0.8303
**Cold~Cold-mild**				
Treatment		3.55	1,19	0.0749
Sex		10.55	1,19	*0.0042*
Female [A]	124.2 ± 1.03			
Male [B]	119.4 ± 1.49			
Treatment:Sex		0.04	1,17	0.8416
**Warm~Warm-mild**				
Treatment:Sex		6.96	1,18	*0.0167*
Warm				
Female [A]	126.7 ± 0.97			
Male [B]	121.6 ± 1.61			
Warm-mild				
Female [A]	124.8 ± 1.43			
Male [A]	132.5 ± 2.24			

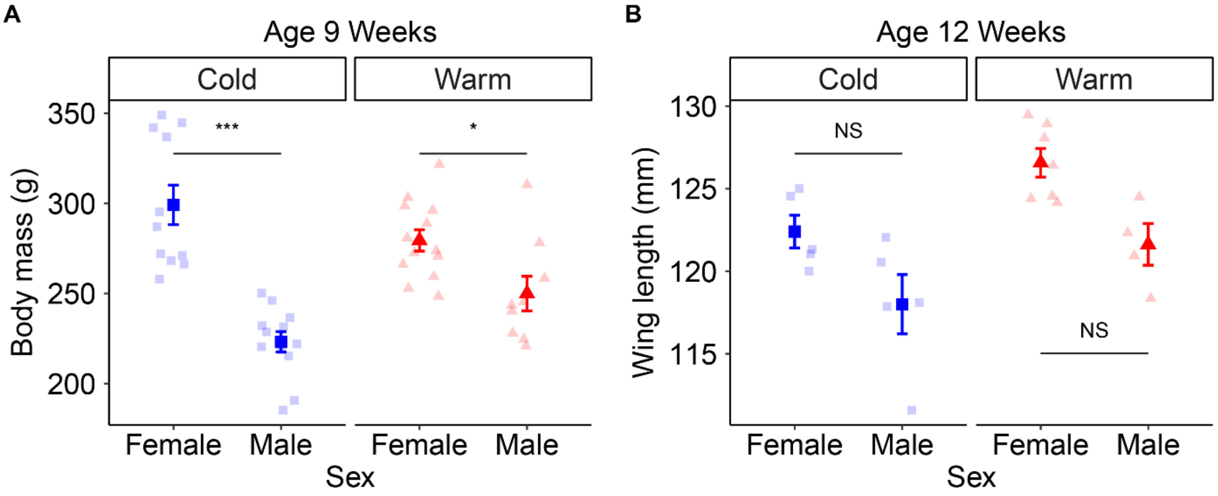

Fig. S6. Differences between sexes within treatments in (A) body mass and (B) wing length of Japanese quail that were raised in either Warm (30°C) or Cold (10°C) conditions (mean ± s.e.). Semi-transparent points show raw data, asterisks represent significance (NS: $P > 0.05$; *: $0.05 \geq P > 0.01$; **: $0.01 \geq P > 0.001$; ***: $P \leq 0.001$). Sample sizes per age and temperature are stated in Table 1.

Journal of Experimental Biology • Supplementary information
